# The Medico-Psychological Association

**Published:** 1918-01-26

**Authors:** H. Wolseley-Lewis, R. H. Cole

**Affiliations:** (Chairman). 10 Chandos Street, Cavendish Square, W. 1; (Secretary). 10 Chandos Street, Cavendish Square, W. 1


					The Medico-Psychological Association.
To the Editor of The Hospital.
Sir,?The treatment of shell-shock and other mental
cases arising from the war has accentuated the necessity
for amendment of the existing Lunacy Laws.
A Select Committee of the Medico-Psychological As-
sociation is now sitting to consider the best method for
securing the legal changes which have been long overdue
in this direction.
The Committee invites information from medical men
and others intimating their experience of the defects in
the present system, and indicating what legislative alter-
ations are in, their opinion desirable.
Communications should be ?ent to the address given
below.
H. Wolseley-Lewis, M.D., F.R.C.S. (Chairman).
R. H. Cole, M.D., iF.R.C.P. . (Secretary).
lu' Ohandos Street, Cavendish Square, W. 1-,
January 19, 1918.

				

